# Urinary C peptide creatinine ratio in pregnant women with normal glucose tolerance and type 1 diabetes: evidence for insulin secretion

**DOI:** 10.1136/bmjdrc-2016-000313

**Published:** 2017-01-04

**Authors:** Ankica Markoska, Rajalakshmi Valaiyapathi, Chloe Thorn, Anne Dornhorst

**Affiliations:** Faculty of Medicine, Department of Investigative Medicine, Hammersmith Hospital, Imperial College London, London, UK

**Keywords:** C-Peptide Physiology, Pregnancy, Type 1, Beta Cell(s)

## Abstract

**Hypothesis:**

In pregnancy, urinary C peptide creatinine ratio (UCPCR) reflects endogenous insulin secretion in women with normal glucose tolerance and type 1 diabetes.

**Research design and methods:**

UCPCR and serum C peptide were measured in 90 glucose-tolerant women at 0 and 120 min during a 75 g oral glucose tolerance test (OGTT) at 28 weeks of gestation. UCPCR was measured in 2 samples obtained over 10 weeks apart in 7 pregnant women with longstanding type 1 diabetes.

**Results:**

UCPCR_OGTT_ and serum C peptide_OGTT_ of glucose-tolerant women were significantly correlated at 0 and 120 min (r_s_0.675, 0.541 respectively, p<0.0001). All 7 pregnant women with type 1 diabetes had detectable first sample UCPCR (median (range) 49 (6–1038) pmol/mmol) that rose in 6 women by 477 (29–1491) pmol/mmol.

**Conclusions:**

Detectable UCPCR in pregnant women with normal glucose tolerance and type 1 diabetes is likely to reflect endogenous insulin secretion and hence β-cell activity.

**Key messages**

- It has previously been shown in the non pregnant state urinary C peptide creatinine ratio (UCPCR) obtained from a spot urine sample correlates with serum C peptide concentration and is a validated method to assess residual β-cell function.
- The current work shows in pregnant glucose tolerant women at 28 weeks gestation UCPCR correlates with serum C peptide at 0 and 120 minutes during a 75 g OGTT
- UCPCR is detectable in pregnant women with over 9 years of type 1 diabetes
- The UCPCR measurement in pregnancy provides a practical method for assessing insulin secretion in pregnancy in women with and without diabetes

## Introduction

Autopsy studies have suggested β-cell proliferation and neogenesis in human pregnancies, possibly due to placental factors.^1–3^ Three studies involving a total of 55 women with type 1 diabetes measured serum or plasma C peptide in pregnancy, showing 49 women to have detectable C peptide values.^4–6^ The ratio of urinary C peptide to the urinary creatinine obtained from a spot urine sample and expressed as UCPCR is correlated to serum C peptide outside pregnancy and has been used to assess residual β-cell function in women with type 1 diabetes. The current study investigated the use of UCPCR to assess β-cell function in pregnant women with normal glucose tolerance and with type 1 diabetes.

## Research design and methods

This prospective study carried out at Queen Charlotte's Hospital was ethically approved by the Imperial College Healthcare Tissue Bank and the Research Ethics Committee Wales: 12/WA/0196. All women gave informed written consent.

One hundred women were recruited prospectively to provide an extra blood and urine sample during a diagnostic 75 g oral glucose tolerance test (OGTT) at 28 weeks of pregnancy for gestational diabetes mellitus (GDM). All women had one or more risk factors for GDM according to the National Institute for Health and Care Excellence (NICE) guidelines,^7^ or were 35 years old or above. All women were fasted for 8–10 hours and had passed their first void morning urine.

Blood samples were collected at 0 (fasting) and 120 min (post-75 g OGTT) in 6 mL BD plastic Vacutainer Plus, silicone coated tubes, placed on ice prior to separation of serum by centrifugation. The second void urine samples at 0 and the 120 min urine sample were collected in 30 mL polystyrene universal containers with boric acid preservative. Serum and urine samples were transferred to cryotubes and stored at −80°C before analysis.

Seven women with previously diagnosed type 1 diabetes were recruited to give a non-fasting spot urine sample in the antenatal clinic on two separate occasions. All seven women gave urine samples that ranged from 10 to 22 weeks apart (five women between the first and third trimester, one between the first and second and one between the second and third trimester). Urine samples were handled as described above.

Urine and serum C peptide was measured by a two-step chemiluminescent microparticle immunoassay using an Abbott Diagnostics Architect platform, with a total coefficient of variation (CV) <10% and a detection range of 3.33–10 000 pmol/L for undiluted samples. Initially urinary c peptide measurements, including those of the seven women with type 1 diabetes, were analyzed undiluted. Samples exceeding the upper limit of detection were rerun following an automated 1:10 dilution using the validated Abbott protein containing diluent. Samples still exceeding the upper limit of detection were rerun following a manual 1:20 dilution using the manufactures' multiassay manual diluent.^8^ Creatinine was measured using the kinetic alkaline picrate method with a total CV of ≤6% (Abbott Architect ci16200 system). The estimated glomerular filtration rate (eGFR) was estimated by the Modification of Diet in Renal Disease (MDRD) formula.

Statistical analysis was performed using SPSS V.22. Correlations between UCPCR_OGTT_ and serum C peptide_OGTT_ at 0 and 120 min for the glucose-tolerant women were performed by Spearman's rank correlation.

## Results

Of the 100 women who had an OGTT, 90 were included for analysis; excluded were 5 women with GDM by NICE criteria,^7^ 2 with non-singleton pregnancies, 2 with a gestational age above 31 weeks and 1 with a renal transplant.

The undiluted OGTT urinary samples were above the upper limit of the C peptide assay detection in 65 of the fasting and in all 90 of the 120 min samples. Following an automated 1:10 dilution, 17 of the 120 min samples remained above the upper range of assay detection. A 1:20 manual dilution was performed on these 17 samples; however, due to technical difficulties during the subsequent analysis and multiple freeze–thaw cycles, these samples were discarded.^9^ Therefore, the analysis of 120 min UCPCR data was performed on the remaining 73 samples.

**Figure 1:**
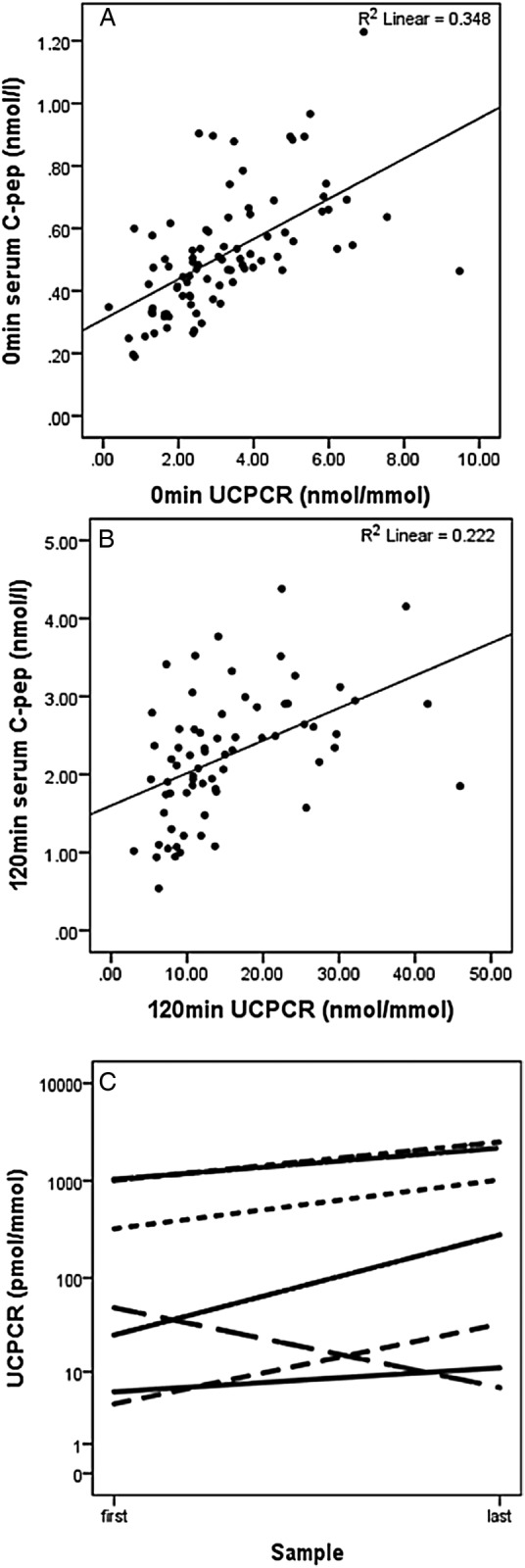
(A) Scatter plot showing the correlation between fasting second void UCPCR and fasting serum C peptide during a 75 g OGTT (r_s_ 0.675, p<0.0001). (B) Scatter plot showing the correlation between 120 min UCPCR and 120 min serum C peptide at 75 g OGTT (r_s_0.541, p<0.0001). (C) The changes in UCPCR of seven pregnant women with type 1 diabetes between two samples taken 10 weeks or more apart. OGTT, oral glucose tolerance test; UCPCR, urinary C peptide creatinine ratio.

The 90 glucose-tolerant women had a median age (range) of 34 (20–49) years, booking body mass index (BMI) of 23.7 (17.96–39.49) kg/m^2^ and a gestational age of 28 (24–29) weeks. The 0 and 120 min serum C peptide_OGTT_ median (25th–75th range) was 483 (381–599) and 2254 (1759–2781) pmol/L, respectively. The UCPCR_OGTT_ at 0 and 120 min median (25th–75th range) were 2796 (1969–3983) and 12 304 (8621–20 733) pmol/mmol, respectively. The UCPCR_OGTT_ and serum C peptide_OGTT_ were significantly correlated at 0 and 120 min, r_s_ 0.675, 0.541 (p<0.0001), respectively (figure 1A, B).

The seven women had previously been clinically diagnosed with type 1 diabetes at a median age of 14.4 (9–25) years. At booking, they were 35 (29–40) years old, with 19 (9–31) years duration of diabetes, BMI of 27.4 (20.3–30.1) kg/m^2^ and glycated hemoglobin of 6.3% (5.4–9.7%; 47 (36–82) mmol/mol). The eGFR of all women was >90 mL/min/1.73 m^2^.

All seven women with type 1 diabetes had detectable postprandial UCPCR in the first and second undiluted urine samples, median (25th–75th range) 173 (5.4–1014) and 650 (27.5–2250) pmol/mmol. Six women had a rise in UCPCR (median rise (25th–75th range) 477 (23–1221) pmol/mmol) whereas one woman had a fall in UCPCR between the two samples collected at 13 and 31 weeks' gestation (figure 1C).

## Discussion

Insulin is secreted in equimolar concentrations as C peptide and UCPCR provides an integrated measurement of insulin secretion over the interval of the urine collection.^10^ As an integrated measure it provides a more informative assessment of insulin secretion over time than a spot serum C peptide concentration that has a 20–30 min circulating half-life.^11^ In addition, the use of the UCPCR provides a more practical methodology due to its ease in collection and processing than serum C peptide in clinical practice and research.^12^

The correlation between UCPCR_OGTT_ and serum C peptide_OGTT_ in 90 glucose-tolerant pregnant women supports the use of the UCPCR to assess insulin secretion during pregnancy. Outside pregnancy, the use of the UCPCR to assess endogenous insulin secretion is well established. The published median (25–75th range) for a mixed meal tolerance test (MMTT) stimulated UCPCR among 27 glucose-tolerant non-pregnant women is 4040 (3000–6990) pmol/mmol; however, this study used a Roche Diagnostic C peptide assay, that using different assay formats and antibodies.^8^ ^13^ These values are approximately a third of the 120 min UCPCR_OGTT_ values of the 73 pregnant glucose-tolerant women (median and 25–75th range of 12 304 (8621–20 733) pmol/mmol) in the current study. An increase in UCPCR and serum C peptide concentration in pregnancy is to be expected due to the physiological decrease in insulin sensitivity that occurs at this time.^14^ The use of the UCPCR in pregnancy should correct for the physiological increase in glomerular filtration rate that occurs throughout pregnancy.^15^

All seven pregnant women with long-term type 1 diabetes had detectable non-fasting UCPCR when first measured, albeit with values approximately a 10th of those seen in the 90 pregnant women with normal glucose tolerance tested at 28 (24–29) weeks' gestation. The published median (25th and 75th range) for a MMTT-stimulated UCPCR of 58 non-pregnant women with type 1 diabetes with over 5 years duration is 20 (0–400) pmol/mmol.^13^ These ranges for UCPCR of non-pregnant women are approximately a fifth lower than those seen for the second urine sample among the seven pregnant women with type 1 diabetes, median (range) 650 (27.5–2250 pmol/mmol), with three women having a UCPCR value >1000 pmol/mmol. However, it has to be recognized that the UCPCR measurements in the current study were performed on undiluted urine that is standard practice in our laboratory for studies in type 1 diabetes. The use of undiluted 24 hours urinary collections has been validated for assaying low C peptide concentrations using the same assay methodology.^8^

Three separate studies in pregnant women with type 1 diabetes have examined circulating C peptide, reporting it either becomes detectable for the first time in pregnancy or increases during pregnancy in some women. Our findings of detectable UCPCR in pregnancy in a small group of women with longstanding type 1 diabetes suggests that using UCPCR in pregnancy might be a suitable methodology for studying pregnancy-induced β-cell regeneration or neogenesis in humans.

There are subtle clinical pointers that pregnancy is related to either β-cell regeneration or neogenesis in women with type 1 diabetes having increased endogenous bioactive insulin secretion. In early pregnancy, there is a decrease of exogenous insulin requirement, and throughout pregnancy a lower than expected incidence of diabetic ketoacidosis despite a fall in serum bicarbonate levels and accelerated maternal lipolysis and ketosis in later pregnancy.^16^

The possibility that residual β-cells in non-pregnant individuals with type 1 diabetes may emerge from neogenesis of pancreatic ductal cells has been suggested.^17^ Somatolactogenic hormones and hyperglycemia have been implicated in the enlargement of the pancreatic islets and β-cell induction and proliferation seen in rodents.^18^

The β-cell adaptation due to neogenesis from other pancreatic cell types, forming new small islets rather than hyperplasia, has been proposed to occur in human pregnancy.^1^ Pregnancy-related factors capable of neogenesis of the human β-cells could have therapeutic implications for the future treatment of type 1 diabetes.

In summary, this study demonstrated that UCPCR provides a robust and practical means for assessing insulin secretion during pregnancy, and provides a practical methodology to assess in future studies the potential for β-cell adaptation in women with type 1 diabetes.
